# Mental health status after living donor hepatectomy

**DOI:** 10.1097/MD.0000000000006910

**Published:** 2017-05-12

**Authors:** Szu-Han Wang, Ping-Yi Lin, Jiun-Yi Wang, Mei-Feng Huang, Hui-Chuan Lin, Chia-En Hsieh, Ya-Lan Hsu, Yao-Li Chen

**Affiliations:** a Organ Transplant Center; b Transplant Medicine & Surgery Research Center, Changhua Christian Hospital, Changhua, Taiwan; c Department of Medical Research, China Medical University Hospital; d Department of Health Care Administration, Asia University; e Department of Nursing, China Medical University Hospital; f Department of Senior Citizen Welfare and Business, Hungkuang University, Taichung, Taiwan; g Transplantation Center, Third Xiangya Hospital of Central South University, Changsha, China; h Department of General Surgery, Changhua Christian Hospital, Changhua; i School of Medicine, Kaohsiung Medical University, Kaohsiung, Taiwan.

**Keywords:** liver transplantation, living liver donor, mental health status

## Abstract

Donor safety and preservation of donor health after living liver donation are of paramount importance. In addition, the preoperative mental state of a donor is an important factor in determining the psychological impact of donor hepatectomy. Thus, we aimed to explore the mental health status of living liver donors after hepatectomy. We enrolled 60 donors who were scheduled to undergo living donor hepatectomy during the period January 2014 to March 2015 at a single medical center. Mental health status was measured before and 3 months after surgery using 3 self-report questionnaires, namely the Center for Epidemiologic Studies Depression Scale (CES-D) to assess depressive symptoms, the World Health Organization Quality of Life (WHOQOL-BREF) questionnaire to measure quality of life, and the Chinese Health Questionnaire (CHQ) to screen for minor psychiatric disorders. A comparison of the pre- and postdonation CES-D scores revealed a significant reduction in depressive symptoms after surgery (*P* = .031). There were significant improvements in the physical health domain (*P* = .031), the psychological health domain (*P* = .005), the social relationships domain (*P* = .005), and the environmental health domain (*P* = .010) of the WHOQOL-BREF. There were no significant changes in CHQ scores after donor hepatectomy (*P* = .136). All donors reported that they would donate again if required. Approximately one-third (33.3%) of donors experienced more pain than they had anticipated in the immediate postoperative period, and 20.0% of donors had complications after donor hepatectomy. Donor mental health status tended to improve as donors regained physical function during the 1st 3 months of recovery. Long-term monitoring of living donors’ mental health is needed to minimize the adverse psychological outcomes of living liver donation.

## 1 Introduction

Liver transplantation is often the only reasonable option for patients with end-stage liver disease.^[1]^ Living donor liver transplantation (LDLT) has become increasingly common worldwide, mainly because of the shortage of cadaveric donor organs but also because of religious and ethical opposition to the practice of deceased-donor transplants, especially in East Asia.^[1]^ According to the literature, the percentage of liver transplants from living donors in East Asia is highest in Japan (99.2%), followed by Korea (65.8%), and Taiwan (36.5%).^[2]^ During the assessment stage, many candidates express concerns about the impact donation will have on their physical health, length of unemployment, their return to daily activities, and their financial status, as well as the outcomes of the recipients.^[3,4]^

A living donor is by definition a healthy person without significant medical problems, and many donors are understandably concerned about the possible consequences of developing postdonation complications that might affect their quality of life, such as biliary complications, abdominal discomfort, and infection.^[5,6]^ Donors also often express concern that they will receive less attention from healthcare providers after surgery than recipients, and studies have documented that donors desire to have a specific individual on the transplant team available to address their postoperative issues.^[7]^

Although many studies have investigated the effects of LDLT on donor quality of life, the impact of LDLT on the postoperative mental health status of donors has not been investigated. Therefore, the purpose of this study was to explore the mental health status of living donors after hepatectomy.

## 2 Patients and methods

### 2.1 Patients

This was a descriptive study based on a cross-sectional survey of living liver donors who underwent donor hepatectomy at a single medical institution in Taiwan. Subjects comprised individuals who were scheduled to undergo donor hepatectomy during the period January 2014 to March 2015 at the Changhua Christian Hospital. Donors were eligible to participate in the study if they were aged ≥18 years, were medically and psychosocially fit to donate, had the ability to understand spoken and written Mandarin Chinese, and agreed to retake the assessment measures 3 months after surgery. A total of 60 participants fulfilled the inclusion criteria and were recruited into the study. Written informed consent was obtained from all donors. Clinical and demographic data were collected from medical records and self-report questionnaires. The study protocol was approved by the Institutional Review Board of Changhua Christian Hospital, and the study was conducted in accordance with the Declaration of Helsinki. Written informed consent was obtained from all donors.

### 2.2 Assessments

Mental health status was measured before and 3 months after surgery using 3 self-report questionnaires, namely the Center for Epidemiologic Studies Depression Scale (CES-D) to assess depressive symptoms, the World Health Organization Quality of Life (WHOQOL-BREF) questionnaire to measure quality of life, and the Chinese Health Questionnaire (CHQ) to screen for minor psychiatric disorders.

### 2.3 Questionnaires

#### 2.3.1 The Center for Epidemiologic Studies Depression Scale (CES-D)

The CES-D was designed to assess depressive symptoms before and after surgery.^[8]^ The CES-D is a 20-item, self-report questionnaire that asks respondents to rate depressive symptoms in the past week using a 4-point scale ranging from 0 (rarely or none of the time) to 3 (most or all of the time). A score of 16 or higher is indicative of clinically significant depressive symptoms.^[9]^ The validity and strong psychometric properties of the CES-D, as well as the high levels of sensitivity and specificity associated with the commonly used cutoff point of ≥16, have been demonstrated in primary care patients with psychological distress based upon typical depressive symptomatology for clinical depression.^[10]^

#### 2.3.2 World Health Organization Quality of Life (WHOQOL-BREF) instrument

The WHOQOL-BREF instrument is an abbreviated self-report questionnaire containing 26 items that measure quality of life. The Chinese version of the WHOQOL-BREF was used in this study. The WHO has determined that the WHOQOL-BREF can be regarded as and used as a cross-cultural questionnaire.^[11]^ The instrument is divided into 4 domains: physical health with 7 items (domain 1), psychological health with 6 items (domain 2), social relationships with 4 items (domain 3), and environmental health with 9 items (domain 4). Each item is rated on a 5-point Likert scale and scored from 1 to 5 on a response scale. Domain scores are scaled in a positive direction, with higher scores indicating better quality of life.

#### 2.3.3 Chinese Health Questionnaire (CHQ)

The CHQ is a self-administered questionnaire designed to screen individuals for the presence of minor psychiatric disorders in the community or in nonpsychiatric departments.^[12]^ The CHQ was modified from the General Health Questionnaire by Cheng and Williams into a 12-item brief psychiatric screening test designed for use in a predominantly Han Chinese population in Taiwan.^[13]^ Participants are asked to respond to 12 items on a 4-point Likert response scale ranging from 1 (not at all) to 4 (a lot more than usual). Higher CHQ scores are indicative of a worse psychiatric state. Cheng et al^[14]^ previously demonstrated that the 12-item CHQ had internal consistency values of 0.84 and 0.83. In the present study, we found that the 12-item CHQ had a Cronbach alpha score of 0.85, indicating a high internal consistency.

### 2.4 Statistical analysis

Continuous variables are presented as means ± standard deviation (SD), and categorical variables are presented as percentages. Differences in questionnaire scores before and after surgery were examined with the McNemar test and paired *t* test to determine the effect of hepatectomy on mental health state. A *P*-value < .05 was considered to represent statistical significance. All statistical analyses were performed on a personal computer with the statistical package SPSS for Windows (version 18, SPSS, Chicago, IL).

## 3 Results

The 60 donors ranged in age from 18 to 62 years (mean age, 30.1 ± 6.8 years). Among them, 46.7% were men, 90% were employed, 63.3% were unmarried, 80.0% donated to an immediate family member, and 90.0% lived with family (Table 1).

**Table 1:**
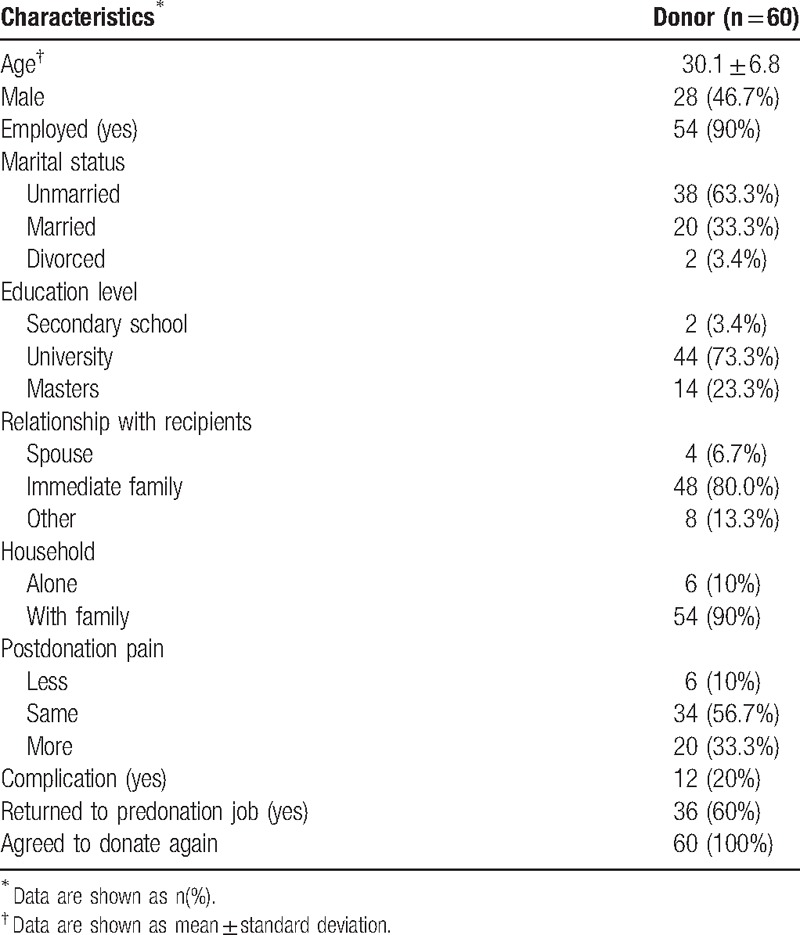
Characteristics of living liver donors.

A comparison of the pre- and postdonation CES-D scores revealed a significant reduction in depressive symptoms after surgery (*P* = .031). In addition, there were significant improvements in the physical health domain (*P* = .031), the psychological health domain (*P* = .005), the social relationships domain (*P* = .005), and the environmental health domain (*P* = .010) of the WHOQOL-BREF. There were no significant changes in CHQ scores after donor hepatectomy (*P* = .136) (Table 2).

**Table 2:**
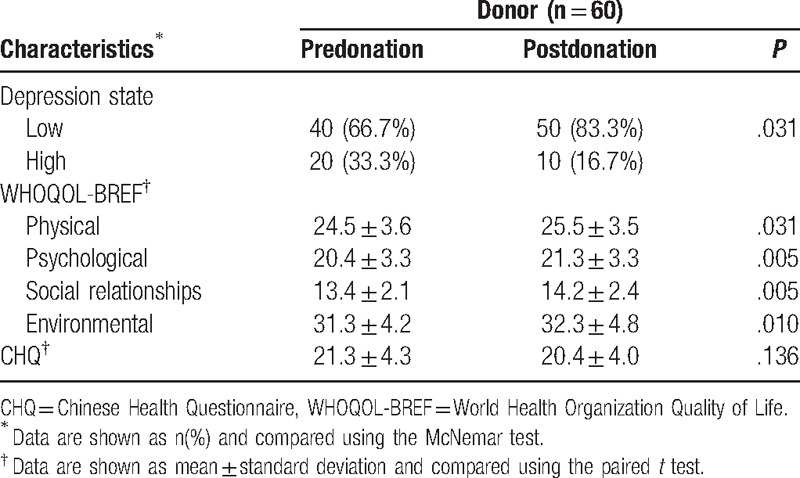
Mental health status of living liver donors.

Postdonation, 20.0% of donors had complications (bile leakage, wound infection, and pleural effusion), and 33.3% experienced more pain than they had anticipated in the immediate postoperative period. Most (60%) donors were able to return to their predonation job within 3 months after surgery, and all donors reported that they would donate again if required (Table 1).

## 4 Discussion

The frequency with which LDLT is performed has increased markedly in the past few decades because of the severe shortage of cadaveric donor organs. Although LDLT has helped to resolve many of the problems faced by patients in need of liver transplant because of end-stage liver disease, the donors themselves achieve no medical benefits and are exposed to risks of surgical complications and negative psychosocial consequences.^[15–17]^ Therefore, donors need to be monitored after surgery not only to assess physical functional recovery but also to detect possible signs of postdonation psychological distress. Studies have shown that approximately 40% of living liver donors report 1 to 3 metrics of psychological distress after surgery and have indicated that those who develop surgical complications score significantly lower on mental and general health scales than donors without major postoperative complications, although most donors still score as well as the general population on mental health scales.^[18–20]^ Chan et al^[21]^ reported that donor quality of life dropped most significantly in the 1st 3 postoperative months, particularly among the physical components of the Karnofsky performance scale, although most patients’ scores returned to preoperative levels within 6 to 12 months.

Most liver donors are young, are in a state of normal psychosocial health, and are optimistic about their future. Although donors understand that the donation surgery can potentially save the recipient's life, the prospect of their career and living arrangements being negatively impacted by the hepatectomy can result in depressive symptoms and a reduction in quality of life leading up to surgery. In our study, donor mental health status tended to improve as donors regained physical function during the 1st 3 months of recovery. Overall, our donors reported a positive experience.

Psychosocial assessments are routinely performed at many living organ donation centers. Such evaluations can identify eligible donors with resilient personality traits and may help to exclude potential donors with a high risk of experiencing psychological problems postoperatively.^[22]^ Social support can mitigate the negative mental states of donors following surgery and has been shown to have a protective effect against depression.^[23]^ Clinicians on the transplantation team should be aware of the kind of support that a donor candidate needs and should provide it in an appropriate manner.^[24]^ According to Taiwan law, donors must undergo a psychiatric evaluation, which includes assessment of mental health and resilience to stress.^[25]^ Resilience has been defined as the ability to recover from setbacks, adapt well to change, and keep going in the face of adversity according to Antonovsky theory of salutogenesis.^[26]^ Resilient persons believe in self-efficacy, have a repertoire of problem-solving skills, and are able to maintain satisfactory interpersonal relationships.^[27]^ Living liver donors have been shown to demonstrate values of resilience comparable to the norm and to have a low level of mental distress.^[24]^ Therefore, for living donors, a high level of mental resilience is a requirement for eligibility.

Studies have shown that most donors return to their predonation job after a mean duration of 3.8 months, and most donors report that they would donate again if necessary.^[28,29]^ In our study, 60% of donors returned to work within 3 months after surgery. However, physical function had yet to return to the preoperative state by 3 months in 40% of donors, which prevented them from returning to work.

Nearly one-third (33.3%) of donors reported that postdonation pain was greater than anticipated in the immediate postoperative period. Moderate to severe pain is a common and anticipated symptom in patients who have undergone hepatectomy for life-threatening indications. Therefore, postsurgery pain in these patients may be overlooked because the surgery is medically required. Conversely, donors are by definition healthy before donation, and thus minor postsurgery discomfort may be more noticeable. Therefore, donors who present with abdominal symptoms after surgery should be closely monitored to determine whether the character or severity of abdominal discomfort changes over time. At our institution, donors and their family members are invited to the transplantation center a week before surgery to receive detailed information regarding the perioperative procedures. We have found that this protocol increases the rate of informed consent during the predonation stage and provides donors and their relatives with a more comprehensive understanding of the surgery. All donors in our study were alive and well at the most recent follow-up, reported that they would donate again if required, and believed that they had benefited from the donation.

The present study has some limitations. First, a self-report questionnaire was used to assess the mental health of donors. Self-reported data are generally reliable and biases due to incorrect recall or unwillingness to reveal personal information are unavoidable. Second, the data were collected at a single medical center in central Taiwan, which may somewhat limit the applicability of the study results. Larger scale studies are required to further verify the findings of the present study. Third, this was a cross-sectional study, so it was not possible to infer causal relationships among the studied variables.

In conclusion, donor mental health status tended to improve as donors regained physical function during the 1st 3 months of recovery. Long-term monitoring of living donors’ mental health is needed to minimize the adverse psychological outcomes associated with living liver donation.
